# Primary Umbilical Endometriosis in an Adolescent Girl: Unsuspected Pathology

**DOI:** 10.1055/s-0039-1700987

**Published:** 2020-02-08

**Authors:** Leel Nellihela, Mudher Al-Adnani, Dorothy Kufeji

**Affiliations:** 1 Department of Paediatric Surgery, Evelina London Children's Hospital, Guy's and St Thomas' NHS Foundation Trust, London, United Kingdom of Great Britain and Northern Ireland; 2 Department of Histopathology, Evelina London Children's Hospital, Guy's and St Thomas NHS Foundation Trust, London, United Kingdom of Great Britain and Northern Ireland

**Keywords:** endometriosis, umbilical, primary, adolescent

## Abstract

Endometriosis affects 7 to 10% of women of reproductive age. Primary umbilical endometriosis (PUE) is even rarer with unclear pathogenesis. We report a case of PUE possibly the youngest patient reported in the literature.

A 16-year-old girl of African origin presented with painful umbilical lump for 2 to 3 months duration with background history of precocious puberty, cyclical vomiting, and menorrhagia. Clinical examination showed dark-colored, tender, irreducible umbilical lump. A provisional diagnosis of incarcerated umbilical hernia was made. Abdominal X-ray showed no features of intestinal obstruction. Ultrasound scan of the abdomen showed lump containing heterogeneous echogenic material measuring 2.0 × 1.5cm within the umbilicus with no visible bowel loops or peristalsis. This was reported as consistent with an umbilical hernia with narrow neck possibly containing mesentery or intra-abdominal fat. The patient underwent urgent exploration of umbilicus under general anesthetic. At operation, a dark-colored, firm mass was excised and sent for histology. The underlying fascia and peritoneum were repaired.

Histological examination confirmed the excised tissue was endometriosis. Follow-up continues in the endometriosis clinic.

Umbilical endometriosis should be considered in differential diagnoses of painful umbilical lesion in adolescent girls and women of reproductive age. Complete excision and histology are highly recommended for obtaining a definitive diagnosis, to exclude malignancy and to prevent recurrence.

## Introduction

Endometriosis is defined as the presence of endometrial glands and stroma outside the uterus. This ectopic finding affects 7 to 10% of women of reproductive age. It commonly occurs in the pelvic organs, presenting with dysmenorrhea, menorrhagia, pelvic pain, and infertility. 1 Ectopic endometrium can occur in the abdominal wall in women as a result of obstetric or gynecologic procedures and also following laparoscopic or other surgical procedures involving the umbilicus (secondary umbilical endometriosis).

Primary umbilical endometriosis (PUE) is the presence of ectopic endometrial tissue in the umbilicus in the absence of previous surgical procedure in that area. PUE is rare and its pathogenesis is unclear. 2 3 4 This is a report of a rare case of PUE in a 16-year-old female that presented as a painful, dark-colored nodule in the umbilicus.

## Case Report

A 16-year-old female patient presented with a painful umbilical lump for a period of 3 months. The lump had been gradually enlarging and was very painful and itchy. It did not respond to two courses of antibiotics, topical steroids, and topical antifungal treatment prescribed by her general practitioner.

She had many medical problems including learning difficulties with autistic spectrum disorder due to a mutation in the TBL1XR1 gene, precocious puberty, obesity (body mass index of 35.4 kg/m ^2^ ), constipation, menorrhagia, and night time bedwetting due to presumed bladder overactivity. She was under endocrinology and urology team review and was treated with Depo-Provera for her menorrhagia. Due to excessive weight gain, Depo-Provera was discontinued and replaced with tranexamic acid.

On physical examination, she had a firm, dark, painful irreducible swelling, 2 cm in diameter, in the umbilicus.

**Fig. 1:**
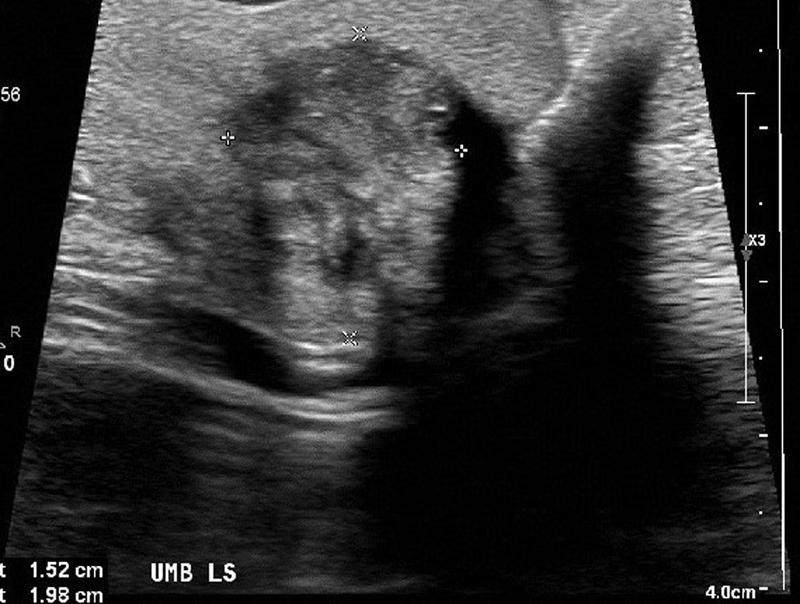
Ultrasonography of the umbilicus demonstrated a 2 × 1.5 cm heterogeneous echogenic material within the umbilicus.

An abdominal X-ray showed a nonspecific bowel gas pattern in the large and small bowel with no features of bowel obstruction. Ultrasonography showed a 2 × 1.5 cm heterogeneous echogenic material within the umbilicus. There was no visible bowel loop or peristalsis within the mass. The appearances were thought to be consistent with an umbilical hernia with narrow neck, possibly containing mesentery or intra-abdominal fat. The lesion was not reducible on examination ( Fig. 1).

The provisional clinical diagnosis was irreducible umbilical hernia with possible strangulated fatty tissue within the hernia sac, and therefore surgical exploration of the umbilicus was performed under general anesthesia with a subumbilical incision.

During exploration, a dark, firm nodule was found within the umbilicus. The nodule was excised with its surrounding tissues consisting of skin, fat, and fascia. Repair of the underlying fascia and peritoneum was performed.

**Fig. 2:**
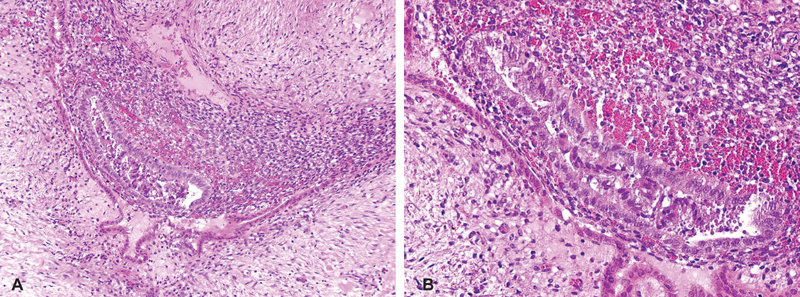
( **A** , **B** ) Histology of the umbilical lesion showing dense fibrosis and glandular structures lined by simple columnar epithelium together with endometrial stroma.

Histopathological examination revealed skin and fatty tissue with dense fibrosis. There were several glandular structures lined by simple columnar epithelium and surrounded by stroma, resembling endometrial stroma. Mild inflammatory changes were found in the surrounding tissue. No granulomas were seen ( Fig. 2 ).

**Fig. 3:**
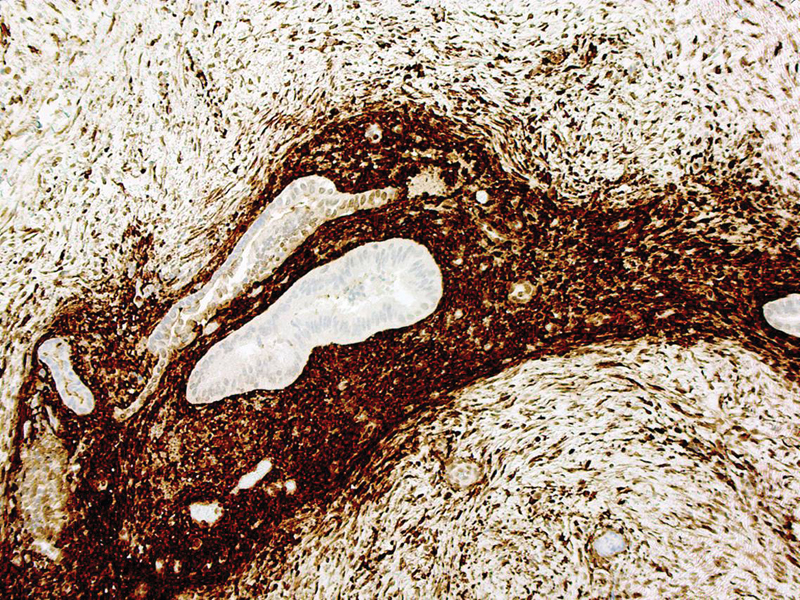
Positive CD10 immunostaining confirmed the presence of endometrial stroma.

CD10 immunostaining confirmed the presence of endometrial stroma, therefore confirming the diagnosis of endometriosis ( Fig. 3 ). Perl's staining showed few hemosiderin laden macrophages.

At 6 months follow-up after the surgery, the patient was asymptomatic with complete healing of the umbilical wound with good cosmetic outcome. She continues to be followed up by the endocrinology team for her weight and menorrhagia.

## Discussion

Endometriosis is defined as the presence of endometrial tissue outside the uterus. The pathogenesis of endometriosis is not known. Possible causes for the development of endometriosis include direct spread, retrograde menstruation, coelomic metaplasia, embryonal rest, and lymphatic or hematogenous spread. Umbilical endometriosis is a rare entity with an estimated incidence of ∼0.5 to 1% of all cases of extragenital endometriosis. 4 However, PUE is an even rarer disorder with very few reported cases in the literature. It has not been reported in a patient as young as our patient (16 years).

The pathogenesis of PUE is not clear. Possible mechanisms include the migration of endometrial cells to the umbilicus through the abdominal cavity, through the lymphatic system, or through the embryonic remnants in the umbilical fold such as the urachus and the umbilical vessels. 5 6 Another possibility is the phenomenon of “coelomic metaplasia.”

Secondary umbilical endometriosis following laparoscopic surgery could result from direct seeding of the endometrium into an umbilical port scar. 7 8 Our patient had no history of laparoscopic surgery in the past, but she had a history of menorrhagia. Whether or not this had any relation to the umbilical endometriosis is unknown. The menorrhagia was treated with Depo-Provera and tranexamic acid. We are not aware of any relationship between the use of these drugs and the development of endometriosis.

Women of reproductive age with umbilical endometriosis present with painful, firm pigmented umbilical nodules associated with cyclic bleeding or discharge during menstruation. The nodule may have a brown, blue, or dark discoloration. 4 There are reported cases where pain may not be associated with menstruation or “cyclic” but “constant” as in our patient.

Umbilical endometriosis should be considered in differential diagnosis of a painful umbilical lesion. Other possibilities include incarcerated hernia, umbilical granuloma, abscess, omphalomesenteric or urachal remnant, and a metastatic malignant lesion.

Imaging modalities such as ultrasonography, computed tomography scan, and magnetic resonance imaging (MRI) are not helpful in establishing a definitive diagnosis of umbilical endometriosis. Ultrasonography can provide some information about the size of the nodule and its adherence to the surrounding tissues. 6 9 For preoperative planning for the excision of the umbilical nodule, MRI is the most accurate method used to assess the depth, especially in relation to the fascial sheath and the peritoneum. This was not performed in this case as the working diagnosis was an umbilical hernia, and umbilical endometriosis was not suspected.

Management of PUE with hormonal therapy using progesterone, danazol, norethisterone, and gonadotropin-releasing hormone analogues has not shown reliable results. However, there are reports of success in relieving symptoms and reducing the size of the endometrial nodule using medical hormonal treatment. 10

Surgical exploration and excision of the nodule should be considered in patients with a symptomatic PUE. The surgical technique should include total excision of the endometriosis lesion, with an adequate rim of normal tissue to avoid local recurrence. It may be necessary to repair the underlying fascia and peritoneum as in our patient. In a patient with PUE, laparoscopy to exclude pelvic endometriosis is a matter of debate. Some authors say it should be avoided in asymptomatic patients as there is a potential risk of introducing endometriosis into the pelvic cavity, 11 while others are in favor of simultaneous laparoscopic exploration to exclude possible further foci of intra-abdominal endometriosis. They argue that pelvic endometriosis cannot be definitively excluded on transvaginal ultrasound or clinical examination. 12 13

Malignant transformation is estimated to occur in 0.6 to 0.8% of cases of ovarian endometriosis. 14 Malignant transformation of umbilical endometriosis has only rarely been reported in the literature. The first reported case of adenocarcinoma of umbilical endometriosis was in 1972. 15 Subsequently in 2013, Obata et al reported a case of clear cell adenocarcinoma arising from umbilical endometriosis in a 60-year-old lady. 16 Koguchi-Yoshioka et al reported a primary adenocarcinoma arising from umbilical endometriosis in a 45-year-old lady in 2016. 14

## Conclusion

PUE is a rare presentation of endometriosis. Complete excision and histology are highly recommended to obtain a definitive diagnosis, exclude malignancy, and prevent recurrence. Laparoscopy to exclude pelvic endometriosis in patients with PUE is debatable.

## Notes

New Insights & the Importance for the Pediatric Surgeon Primary umbilical endometriosis should be considered in differential diagnosis in adolescent girls presented with painful umbilical lesion. Complete excision is prerequisite to prevent recurrence.
